# International Consensus Recommendations for the Treatment of Pediatric NMDAR Antibody Encephalitis

**DOI:** 10.1212/NXI.0000000000001052

**Published:** 2021-07-22

**Authors:** Margherita Nosadini, Terrence Thomas, Michael Eyre, Banu Anlar, Thais Armangue, Susanne M. Benseler, Tania Cellucci, Kumaran Deiva, William Gallentine, Grace Gombolay, Mark P. Gorman, Yael Hacohen, Yuwu Jiang, Byung Chan Lim, Eyal Muscal, Alvin Ndondo, Rinze Neuteboom, Kevin Rostásy, Hiroshi Sakuma, Suvasini Sharma, Silvia Noemi Tenembaum, Heather Ann Van Mater, Elizabeth Wells, Ronny Wickstrom, Anusha K. Yeshokumar, Sarosh R. Irani, Josep Dalmau, Ming Lim, Russell C. Dale

**Affiliations:** From the Paediatric Neurology and Neurophysiology Unit (M.N.), Department of Women's and Children's Health, University Hospital of Padova; Neuroimmunology Group (M.N.), Paediatric Research Institute “Città della Speranza,” Padova, Italy; Department of Paediatrics (T.T.), Neurology Service, KK Women's and Children's Hospital, Singapore; School of Biomedical Engineering & Imaging Sciences (M.E.), King's College London; Children's Neurosciences (M.E.), Evelina London Children's Hospital at Guy's and St Thomas' NHS Foundation Trust, United Kingdom; Department of Pediatric Neurology (B.A.), Hacettepe University, Ankara, Turkey; Neuroimmunology Program (T.A.), Institut d'Investigacions Biomèdiques August Pi i Sunyer (IDIBAPS), Hospital Clínic, Universitat de Barcelona; Pediatric Neuroimmunology Unit (T.A.), Neurology Department, Sant Joan de Déu (SJD) Children's Hospital, University of Barcelona, Spain; Alberta Children's Hospital Research Institute (S.M.B.), Department of Pediatrics, Cumming School of Medicine, University of Calgary; Division of Rheumatology (T.C.), Department of Pediatrics, McMaster University, Hamilton, Ontario, Canada; Assistance Publique-Hôpitaux de Paris (K.D.), Pediatric Neurology Department, University Hospitals Paris Saclay, Bicêtre Hospital, France; French Reference Network of Rare Inflammatory Brain and Spinal Diseases (K.D.), Le Kremlin Bicêtre, France and European Reference Network-RITA; Departments of Neurology and Pediatrics (W.G.), Stanford University and Lucile Packard Children's Hospital, Palo Alto, CA; Division of Pediatric Neurology (G.G.), Department of Pediatrics, Emory University School of Medicine and Children's Healthcare of Atlanta, GA; Department of Neurology (M.P.G.), Boston Children's Hospital, Harvard Medical School, Boston, MA; Department of Neuroinflammation (Y.H.), Queen Square MS Centre, UCL Institute of Neurology, University College London; Department of Paediatric Neurology (Y.H.), Great Ormond Street Hospital for Children, London, United Kingdom; Department of Pediatrics (Y.J.), Peking University First Hospital, Beijing, China; Department of Pediatrics (B.C.L.), Pediatric Clinical Neuroscience Center, Seoul National University Children's Hospital, Seoul National University College of Medicine, South Korea; Department of Pediatrics (E.M.), Section Rheumatology, Co-appointment in the Section of Neurology and Developmental Neuroscience, Texas Children's Hospital, Baylor College of Medicine, Houston; Division of Paediatric Neurology (A.N.), Department of Paediatrics and Child Health, Red Cross War Memorial Children's Hospital, University of Cape Town; Faculty of Health Sciences (A.N.), University of Cape Town Neuroscience Institute, South Africa; Department of Neurology (R.N.), Erasmus Medical Center, Rotterdam, the Netherlands; Department of Pediatric Neurology (K.R.), Children's Hospital Datteln, University Witten/Herdecke, Germany; Department of Brain and Neural Science (H.S.), Tokyo Metropolitan Institute of Medical Science, Japan; Department of Pediatrics (Neurology Division) (S.S.), Lady Hardinge Medical College and Associated Kalawati Saran Children's Hospital, New Delhi, India; Department of Neurology (S.N.T.), National Pediatric Hospital Dr. J. Garrahan, Buenos Aires, Argentina; Department of Pediatrics (H.A.V.M.), Duke University, Durham, NC; Department of Neurology (E.W.), Children's National Medical Center, Washington, DC; Neuropaediatric Unit (R.W.), Karolinska University Hospital, Stockholm, Sweden; Department of Neurology (A.K.Y.), Icahn School of Medicine at Mount Sinai, New York; Oxford Autoimmune Neurology Group (S.R.I.), Nuffield Department of Clinical Neurosciences, University of Oxford, John Radcliffe Hospital; Department of Neurology (S.R.I.), Oxford University Hospitals NHS Foundation Trust, United Kingdom; Neuroimmunology Program (J.D.), Institut d'Investigacions Biomèdiques August Pi i Sunyer (IDIBAPS), Hospital Clínic, University of Barcelona, Spain; Department of Neurology (J.D.), University of Pennsylvania, Philadelphia; Institució Catalana de Recerca i Estudis Avançats (ICREA) (J.D.), Barcelona, Spain; Children's Neurosciences (M.L.), Evelina London Children's Hospital at Guy's and St Thomas' NHS Foundation Trust; King's Health Partners Academic Health Science Centre (M.L.); Faculty of Life Sciences and Medicine (M.L.), King's College Hospital, United Kingdom; and Kids Neuroscience Centre (R.C.D.), The Children's Hospital at Westmead, Faculty of Medicine and Health, University of Sydney, NSW, Australia.

## Abstract

**Objective:**

To create an international consensus treatment recommendation for pediatric NMDA receptor antibody encephalitis (NMDARE).

**Methods:**

After selection of a panel of 27 experts with representation from all continents, a 2-step Delphi method was adopted to develop consensus on relevant treatment regimens and statements, along with key definitions in pediatric NMDARE (disease severity, failure to improve, and relapse). Finally, an online face-to-face meeting was held to reach consensus (defined as ≥75% agreement).

**Results:**

Corticosteroids are recommended in all children with NMDARE (pulsed IV preferred), with additional IV immunoglobulin or plasma exchange in severe patients. Prolonged first-line immunotherapy can be offered for up to 3–12 months (oral corticosteroids or monthly IV corticosteroids/immunoglobulin), dependent on disease severity. Second-line treatments are recommended for cases refractory to first-line therapies (rituximab preferred over cyclophosphamide) and should be considered about 2 weeks after first-line initiation. Further immunotherapies for refractory disease 1-3 months after second-line initiation include another second-line treatment (such as cyclophosphamide) and escalation to tocilizumab. Maintenance immune suppression beyond 6 months (such as rituximab redosing or mycophenolate mofetil) is generally not required, except for patients with a more severe course or prolonged impairments and hospitalization. For patients with relapsing disease, second-line and prolonged maintenance therapy should be considered. The treatment of NMDARE following herpes simplex encephalitis should be similar to idiopathic NMDARE. Broad guidance is provided for the total treatment duration (first line, second line, and maintenance), which is dictated by the severity and clinical course (i.e., median 3, 9 and 18 months in the best, average, and worst responders, respectively). Recommendations on the timing of oncologic searches are provided.

**Conclusion:**

These international consensus recommendations for the management of pediatric NMDARE aim to standardize the treatment and provide practical guidance for clinicians, rather than absolute rules. A similar recommendation could be applicable to adult patients.

NMDA receptor antibody encephalitis (NMDARE) is one of the most common autoimmune encephalitides, characterized by a recognizable constellation of neurologic and psychiatric features alongside positive NMDAR antibodies.^1,2^ NMDARE mostly affects children and young adults, particularly females. It may be very severe in the acute phase with a mortality of about 5%, relapses occur in about 15% of patients, and the final physician-assessed functional outcome is generally favorable, although neuropsychological and psychiatric sequelae are relatively common.^2,3^

The use of immunotherapies has been shown to improve outcomes,^2,4-6^ especially with early administration.^2,4,6,7^ In addition, immunotherapies reduce the risk of relapses.^2,8,9^ However, several aspects of treatment remain incompletely clarified, and treatment strategies are still heterogeneous, especially with regard to second-line and long-term immunotherapies.^10,11^ Indeed, although a number of reviews have been published,^12-18^ no randomized controlled trials or consensus guidelines for the treatment of NMDARE are available.

With support from the Autoimmune Encephalitis Alliance, we aimed to create a consensus recommendation for the treatment of pediatric NMDARE, which was pragmatic and relevant to a global community and could serve as a practical decision support tool for the clinician confronted with this rare and challenging condition. Notably, the present document is intended as a recommendation guideline rather than absolute rules, given the limited evidence supporting most treatment statements. Although this document is focused on immunotherapy and to some extent symptomatic management, there are multiple outstanding issues in the management of pediatric NMDARE, such as education around the diagnosis and rehabilitation of patients after the acute phase, which are beyond the scope of this current article.

## Methods

### Establishment of a Consensus Expert Panel

A steering committee (R.C.D., M.L., T.T., M.N., and M.E.) carefully selected a panel of 27 experts with representation from all continents (later referred to as “the Panel”), and based on the individual: (1) being a specialist (usually pediatric neurologist or rheumatologist) with clinical and/or research expertise in pediatric NMDARE; these experts were identified as lead clinical researchers in the field based on the systematic review conducted before the consensus recommendations project (paper in preparation), or were nominated by national child neurology societies; (2) having a publication track record in the field of pediatric autoimmune encephalitis/CNS disease; (3) being committed to completing 2 Delphi studies (approximately 45 minutes each),^19,20^ and participating in a 2-hour face-to-face/online meeting to reach consensus. The 27 experts were pediatric neurologists (n = 23) or pediatric rheumatologists (n = 4), from North America (n = 9), South America (n = 1), Europe (n = 9), Asia (n = 6), Oceania (n = 1), and Africa (n = 1). In addition, patient representatives (parents, n = 2), a member of the Autoimmune Encephalitis Alliance (n = 1), and adult neurology experts in NMDARE (n = 2, J.D. and S.R.I.) were invited to provide input in the later stages of the process.

### Delphi Method

A 2-step Delphi method was adopted to develop the consensus of relevant statements, similar to the method used by the European League Against Rheumatism.^21^ A document with key definitions in pediatric NMDARE (disease severity, failure to improve, and relapse) used in the Delphi statements was shared online with the Panel (January 2020) before the first Delphi questionnaire. A revised version of the modified Rankin Scale^22^ was used, to be more applicable in children.

The first Delphi questionnaire (Delphi 1, eAppendix 1, links.lww.com/NXI/A530) included key statements on the treatment of pediatric NMDARE, which were created based on the steering committee's clinical practice and the available literature and was sent out to the Panel in February 2020 using a web-based survey tool (SurveyMonkey.com). The Panel members were asked to vote on each statement of the first Delphi questionnaire according to a 5-point Likert scale (strongly agree/agree/neither agree nor disagree/disagree/strongly disagree) and provide open text comments as appropriate. Consensus was defined as an agreement by at least 75% of the participants (i.e., ≥75% agree/strongly agree or ≥75% disagree/strongly disagree).

Twenty-six of 27 experts completed Delphi 1; then, the statements were revised according to the Panel's responses and comments, and statements that reached consensus were collated into a second Delphi document (Delphi 2). In this second Delphi survey, time durations were added (i.e., total duration of immunotherapy in NMDARE or timing of treatment escalation), and median, interquartile range (IQR), and range were calculated. The Delphi 2 statements were shared with 2 adult experts (J.D. and S.R.I.), with the Autoimmune Encephalitis Alliance representative and family representatives for further input. Delphi 2 was completed by 26 of the 27 experts by online survey in May 2020 (eAppendix 1, links.lww.com/NXI/A530), and final drafted recommendations were created.

### Face-to-Face Meeting

The drafted recommendations were then voted on during a 2-hour online consensus meeting via the platform Zoom (zoom.us) on November 3, 2020, and included 26 participants from the expert Panel, with representatives from all continents. Each recommendation was voted on via the platform sli.do with the outcomes agree, do not agree, or abstain. The definitions used in the recommendations and the drug regimens were also voted on for consensus. As before, consensus was defined as an agreement by at least 75% of the participants.

The number of voters varied (22-26 panelists) for the statements due to connectivity issues during the meeting. The statements that reached consensus were collated and are presented.

### Data Availability

The Delphi questionnaires used to create the consensus-based recommendations for the treatment of pediatric NMDARE are provided in eAppendix 1 (links.lww.com/NXI/A530).

## Results

eAppendix 1 (links.lww.com/NXI/A530) provides the Delphi 1 and Delphi 2 questionnaires and answers. Only final recommendations that reached consensus at the final face-to-face meeting are presented in Tables 1–4 and the Figure. Table 1 shows the key definitions in pediatric NMDARE (disease severity, failure to improve, and relapse), which reached consensus support. In addition, to aid clinicians with less experience in the management of NMDARE, definitions for best, average, and poorest responders are described (Table 5). Tables 2 and 3 show the recommendations for the treatment of pediatric NMDARE and are subdivided into general management principles (Table 2, 2.1), treatment of first encephalitis event including first-line, second-line, and maintenance immunotherapy (Table 2, 2.2–2.4), overall duration of immunotherapy at first event (Table 2, 2.5), treatment at relapse (Table 3, 3.1), treatment of NMDARE triggered by preceding herpes simplex virus encephalitis (HSE) (Table 3, 3.2), symptomatic treatments (Table 3, 3.3), and oncologic searches (Table 3, 3.4). Table 4 shows the recommendations for immunotherapy doses and regimens.^23-25^ The Figure provides a therapeutic pathway for guidance.

**Table 1:**
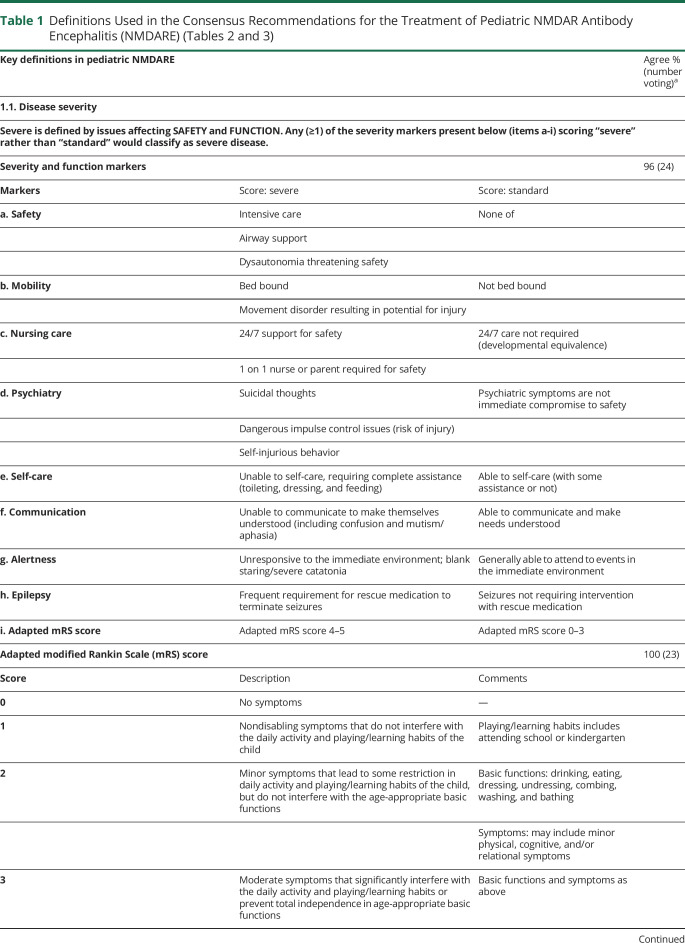

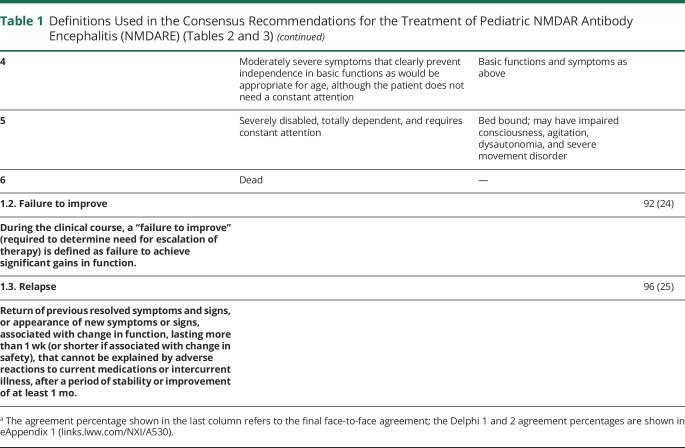
Definitions Used in the Consensus Recommendations for the Treatment of Pediatric NMDAR Antibody Encephalitis (NMDARE) (Tables 2 and 3)

**Table 2:**
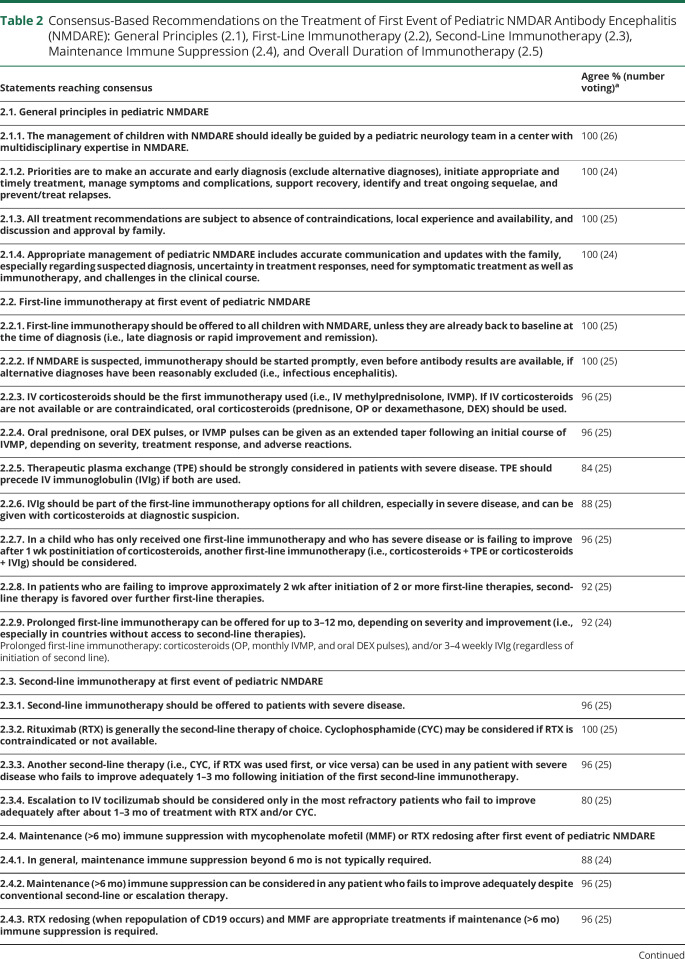

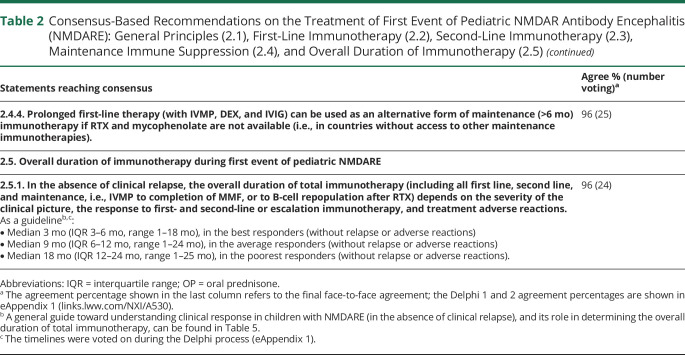
Consensus-Based Recommendations on the Treatment of First Event of Pediatric NMDAR Antibody Encephalitis (NMDARE): General Principles (2.1), First-Line Immunotherapy (2.2), Second-Line Immunotherapy (2.3), Maintenance Immune Suppression (2.4), and Overall Duration of Immunotherapy (2.5)

**Table 3:**
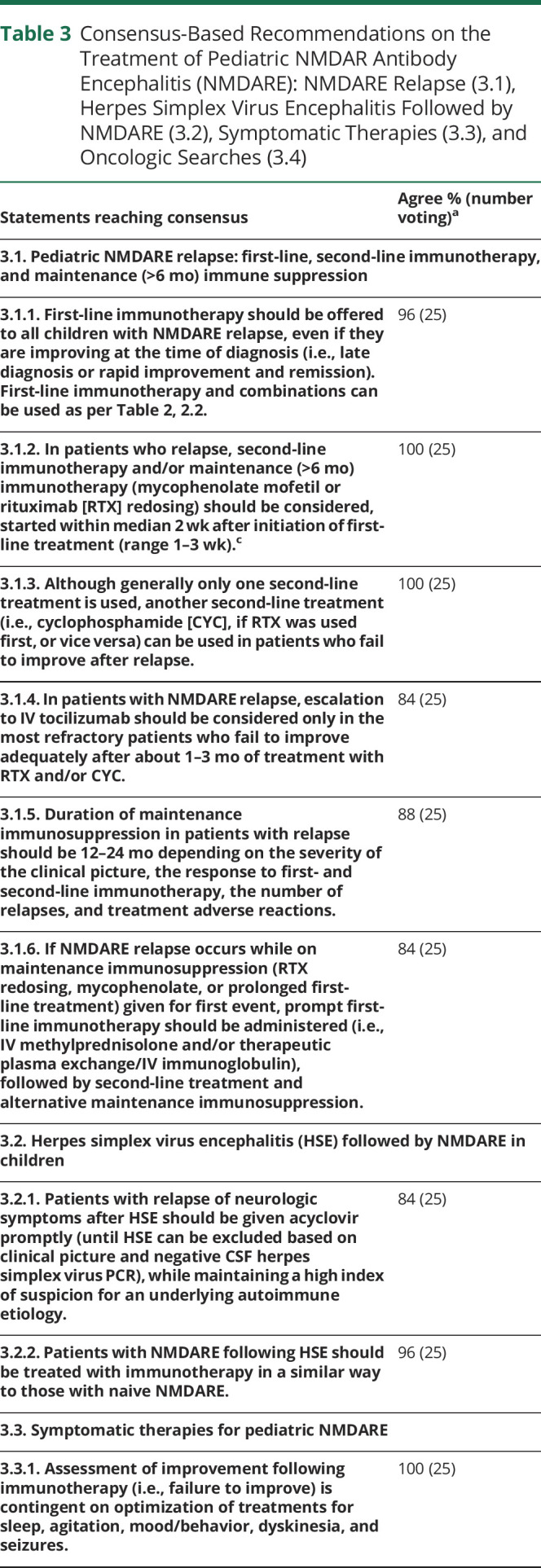

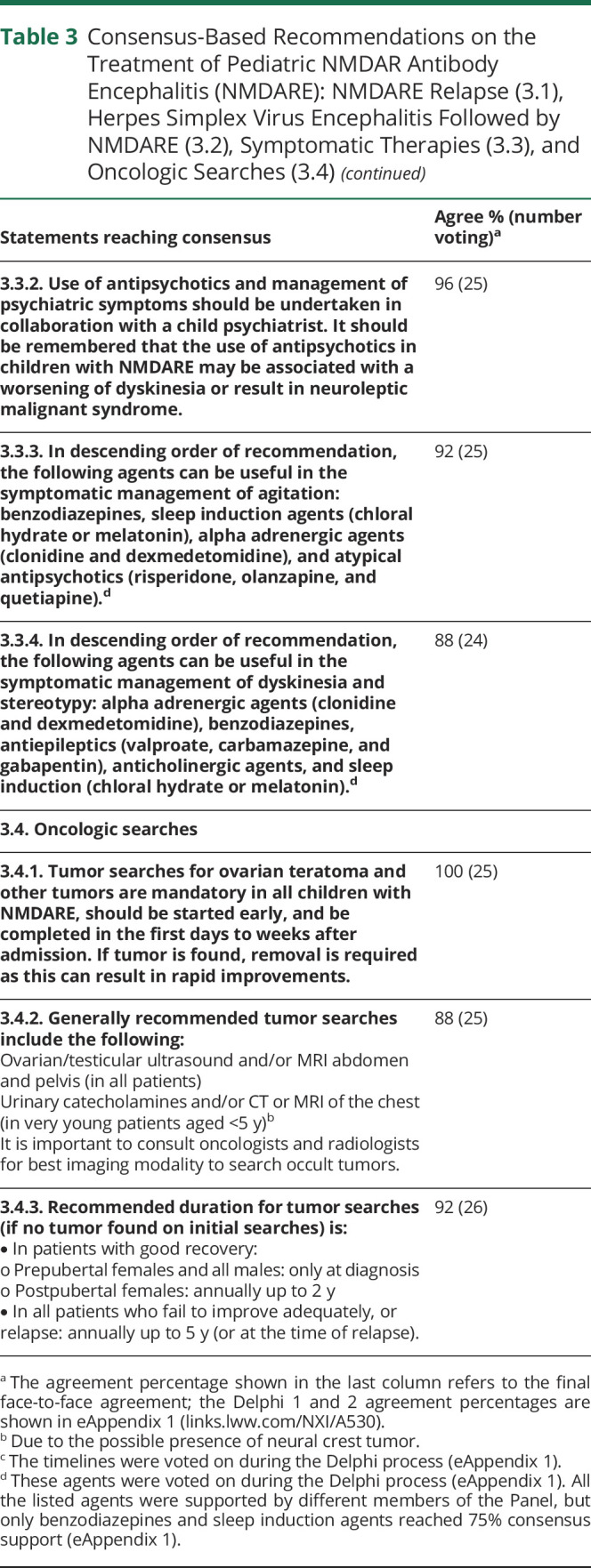
Consensus-Based Recommendations on the Treatment of Pediatric NMDAR Antibody Encephalitis (NMDARE): NMDARE Relapse (3.1), Herpes Simplex Virus Encephalitis Followed by NMDARE (3.2), Symptomatic Therapies (3.3), and Oncologic Searches (3.4)

**Table 4:**
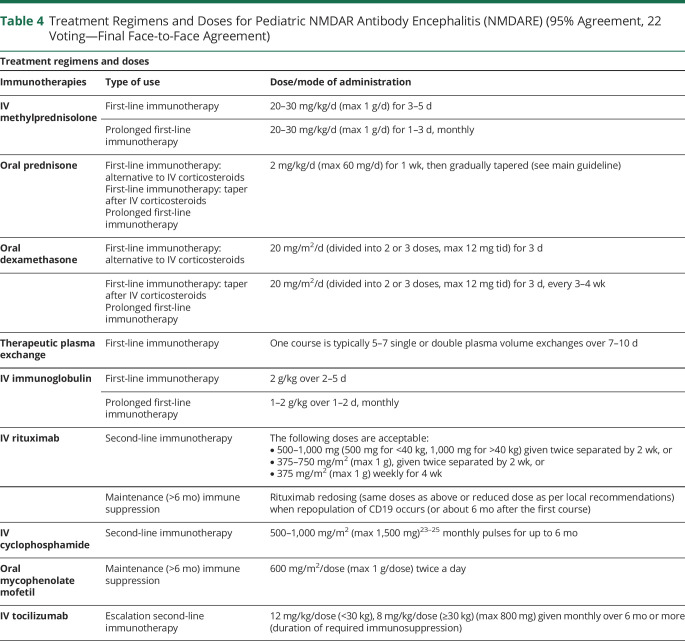
Treatment Regimens and Doses for Pediatric NMDAR Antibody Encephalitis (NMDARE) (95% Agreement, 22 Voting—Final Face-to-Face Agreement)

**Figure:**
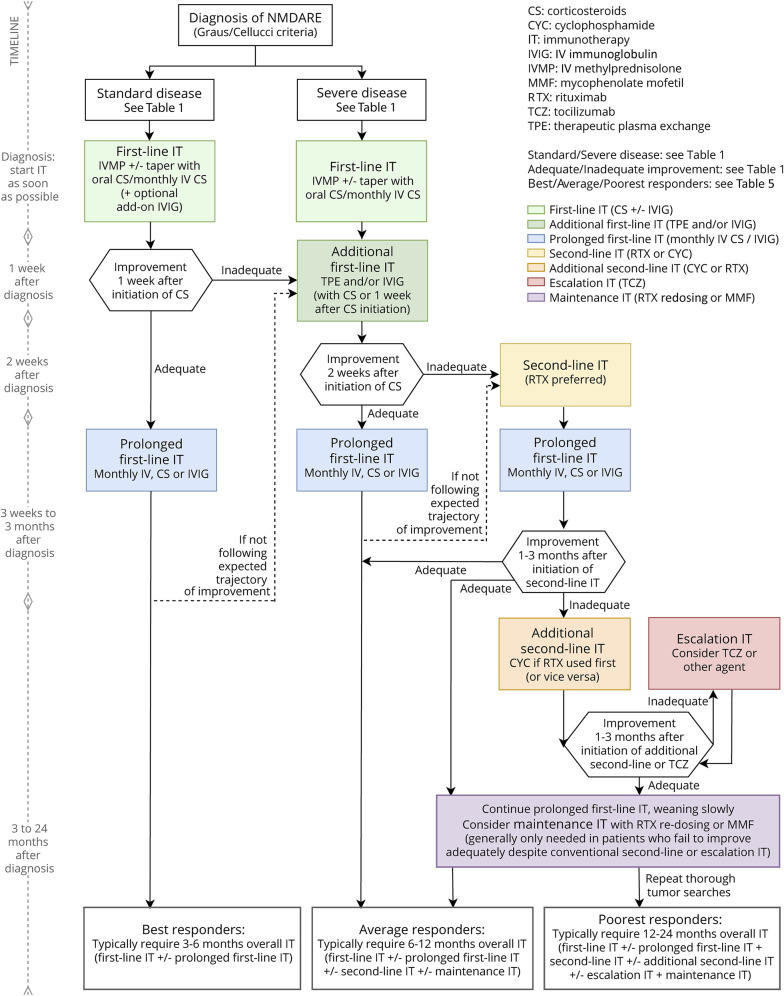
International Consensus Recommendations for the Treatment of First Event of Pediatric NMDAR Antibody Encephalitis (NMDARE)

**Table 5:**
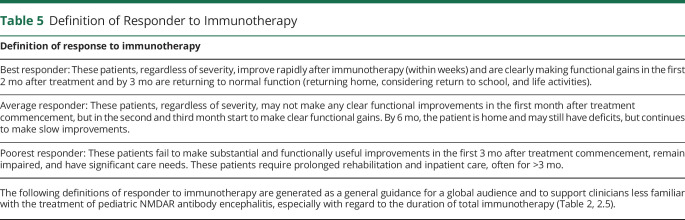
Definition of Responder to Immunotherapy

## Discussion

Evidence on treatment of NMDARE is restricted to retrospective and some prospective descriptive studies. No consensus-based treatment guidelines have previously been proposed. Hence, our purpose was to create international consensus-based recommendations for the treatment of pediatric NMDARE, with expertise from an international group of clinical and academic pediatric neurologists and rheumatologists. Our vision was to have a global approach with applicability across all health care settings; therefore, the expert Panel included representatives from all continents. We also wanted this document to be useful for clinicians less experienced in the treatment of autoimmune encephalitis; hence, a practical and detailed approach was adopted wherever possible, including definitions of failure to respond, and timing of treatment escalation. Indeed, although the management of pediatric NMDARE should ideally be guided by a pediatric neurology team in a center with multidisciplinary expertise in NMDARE, this may not always be possible, particularly in the acute phase of the disease.

Our recommendations begin with general management principles, highlighting the importance of early diagnosis and careful communication with the family (Table 2, 2.1). The importance of raising awareness of this disorder, which may present to psychiatrists and emergency physicians as well as neurologists, cannot be overemphasized, and the diagnostic criteria^26^ and modification for children,^27^ along with the distinctive clinical characteristics,^12,28,29^ may aid an expeditious diagnosis. Similarly, families need to be informed of the expected or potential disease evolution, the treatment possibilities, and the often long and demanding course of the illness. Understanding the timeline of the disease and the speed of recovery is one of the greatest challenges of this disease, and it is essential for clinicians and family members to appreciate that the typical course is of little change (or worsening) in the first weeks and slow improvements in the following months, and improvements may continue into the second year.

As regards first-line immunotherapy (Table 2, 2.2), there was consensus that corticosteroids are the first agent to be used in pediatric NMDARE, with IV use (i.e., IV methylprednisolone) preferred over oral use (i.e., oral prednisone), although high-dose oral administration of corticosteroids is a good alternative, particularly if IV access is a problem. In high-income countries, therapeutic plasma exchange (TPE) and/or IV immunoglobulin (IVIg) are often used in conjunction with corticosteroids.^30^ Although some physicians use TPE or IVIg at the same time as corticosteroids, other administer them sequentially, with more severe patients often prompting a more aggressive combined treatment or rapid escalation. TPE was recommended for patients with severe disease, although it is recognized that TPE can be associated with more severe complications (e.g., central line infection) compared with IVIg.^31,32^ TPE was recommended over immunoadsorption, where there is less evidence.^33,34^ In general, ongoing corticosteroids are continued in the first months of disease, preferably as pulses, or alternatively oral tapers. Longer or repeated IVIg courses may be continued monthly for 3–6 months, depending on severity and availability, whereas monthly pulsed oral dexamethasone or IV methylprednisolone, or even ACTH, for 3-6 months may be used in resource-limited settings.

In patients who are failing to improve (definition in Table 1) approximately 2 weeks after initiation of 2 or more first-line therapies, second-line treatment is recommended over further first-line therapies. Second-line treatments are recommended especially in patients with severe disease, with rituximab now generally preferred over cyclophosphamide (Table 2, 2.3). Rituximab dosing protocols were all equally accepted (Table 4) as there are no data to support one protocol over another. There is evidence suggesting that use of second-line immunotherapy improves outcome in patients failing to improve after first-line therapy^2^ and that second-line therapy reduces the risk of relapses.^8,9,13^ Moreover, earlier initiation of rituximab also seems more favorable compared with late treatment.^7^

The use of second-line immunotherapy is still variable globally and considerably less frequent in some countries. For instance, rituximab use is 0%–5.5% in Chinese cohorts^35-37^ and more variable in India (0%–61%),^38-40^ although with generally favorable outcomes, which suggests the outcomes described in the published literature may be affected by referral bias, publication bias, or ethnic vulnerability to worse outcomes.^41^ The specific approaches toward the use of second-line immunotherapy varied within the Panel, with some clinicians supporting the use of rituximab in all patients with NMDARE and others reserving it to cases with severe disease or failure to improve (Table 1). The consensus opinion was that second-line therapy is not needed in all patients, but only in patients with severe disease and those who fail to improve.

One of the greatest challenges is deciding the timing of escalation after 1 second-line therapy. There was consensus that in the patient failing to improve 1-3 months (generally >6 weeks) following initiation of the first second-line immunotherapy, another second-line therapy such as cyclophosphamide if rituximab was used first can be considered.

In the patient who fails rituximab, cyclophosphamide is generally recommended as an escalation agent, although some members of the Panel have increasing interest in tocilizumab as an alternative escalation therapy due to a more favorable perceived safety profile.^42-44^ Other escalation treatments have been reported in the literature, such as IV/intrathecal methotrexate with intrathecal corticosteroids and subcutaneous/IV bortezomib; these have more limited evidence, but can be used according to the local treating center's expertise.^41,43-57^

The patient who has severe disease and is failing to improve remains a major challenge. The clinician needs to balance the risk of severe disease (such as being on the intensive care unit) with the risk of treatment side effects, in the knowledge that NMDARE symptoms may take many weeks or months to improve.^2,7^ Indeed, unlike in acute disseminated encephalomyelitis, when treatment often results in rapid improvements within days, in NMDARE, the improvements are slow and continue for ≥24 months after the acute phase.^2^ Therefore, allowing treatments to have their effect, including their combined actions, is important to avoid hasty therapeutic decisions. In general, second-line agents such as rituximab or cyclophosphamide should be given 1-3 months before making judgment on effect, with 6 weeks being a broadly accepted guideline. The timing of escalation is very challenging and influenced by severity, age, risk-benefit ratio, treating center's experience, and access to treatments. Overall, for patients in the intensive care unit, where there may be multiple additional risk factors,^7^ earlier escalation seems reasonable. Anecdotal reports from our expert group of benefit of treatment with rituximab or tocilizumab years after onset suggest that in the patient who continues to have major impairments, further immunotherapies are warranted within reason, although there are likely to be diminishing returns when treatment is used later in the disease course.

In the patient who has failed to improve a year or more after treatment, it is sometimes difficult to determine residual sequelae from ongoing inflammation. In this situation, CSF re-examination for ongoing neuroinflammation (i.e., persistent pleocytosis, intrathecal oligoclonal bands, elevated immunoglobulin G [IgG] index, or CSF neopterin)^58^ may help with decision making and the risk vs benefit consideration of an empiric retrial or immunotherapy (pulsed corticosteroid for 3 months, IVIg monthly, rituximab reinduction, or tocilizumab). CSF NMDAR antibody titers seem to correlate better with disease course compared with serum antibodies,^59,60^ but there is not a strong correlation between titer and clinical course in the individual patient, and antibodies can persist long after recovery.^60,e1,e2^ Although all stages of management of NMDARE may be challenging even for experienced physicians, this is especially true when dealing with a severe patient failing to improve, and a second opinion may be useful and help the clinician make further therapeutic decisions. Organizations such as the Autoimmune Encephalitis Alliance (aealliance.org/), the Encephalitis Society (encephalitis.info/), and the Anti NMDA Receptor Encephalitis Foundation Inc. (antinmdafoundation.org/) may help connect with experts.

There was overall agreement that maintenance immune suppression beyond 6 months from onset is generally not needed (Table 2, 2.4), apart from patients with more severe course or prolonged impairments and hospitalization. Indeed, literature data show that early and adequate treatment, including use of second-line therapies when appropriate, is the priority,^2^ rather than prolonged maintenance immune suppression. Moreover, the relatively low relapse rate of NMDARE is in significant contrast with that of other disorders such as neuromyelitis optica, where chronic immune suppression is recommended from the first event. When giving immune suppression for more than 6 months, rituximab redosing was generally preferred, although mycophenolate mofetil is also used,^9,36,e3-e5^ and there is little evidence to suggest superiority of either. With regard to rituximab redosing, most experts recommend redosing when CD19 cells repopulate, in view of the variability in the time to B-cell repopulation between individuals.^e6^ An alternative approach is to redose rituximab at regular 6-month intervals similar to practice in adult patients with neuromyelitis optica.^e7,e8^ There was no consensus in the dosage and frequency of redosing, with some experts using the same dose/regimen used at induction and others using lower doses (Table 4). As regards mycophenolate mofetil, given its slow onset of efficacy, there should initially be overlap with other immunotherapies (i.e., oral corticosteroids) for 3-6 months after commencement.^e3^ Other maintenance agents, such as oral azathioprine and methotrexate, are sometimes used for maintenance immune suppression, although the paucity of experience precluded consensus recommendations from our expert group. In resource-poor countries, the Panel also agreed that prolonged first-line therapy (with IV pulsed methylprednisolone, dexamethasone, or IVIg) can be used as an alternative form of maintenance (>6 months) immunotherapy, if rituximab and mycophenolate mofetil are not available.

There was agreement in the need for a more aggressive and prolonged treatment approach in patients with relapsing disease (Table 3, 3.1), with a lower threshold for second-line and maintenance treatments (rituximab or mycophenolate) and more prolonged overall immunotherapy duration. Indeed, the median overall duration of immunotherapy at first event of pediatric NMDARE was recommended to be about 3 months (IQR 3–6 months) in the best responders, 9 months (IQR 6–12 months) in the average responders, 18 months (IQR 12–24 months) in the poorest responders (Table 2, 2.5), and 12–24 months after a relapse, acknowledging patient severity and management variables (Table 3, 3.1). We acknowledge that the definition of “best,” “average,” and “poorest” is dependent on experience of the clinician; therefore, some guidance is provided in Table 5.

Although not the focus of this work, the Panel acknowledges that infectious risk mitigation strategies are key to ensure the patients' safety while receiving immunotherapy, especially close monitoring for infections and adherence to hospital infection control protocols to prevent hospital acquired infection. In selected patients on prolonged high-dose corticosteroids, multiple second-line or escalation immunotherapies, prophylactic trimethoprim-sulfamethoxazole for *Pneumocystis carinii* pneumonia may be required. In patients with low IgG levels and recurrent infections despite prophylactic antibiotics, immunoglobulin supplementation may be required.

As regards patients with relapse of neurologic symptoms after HSE (Table 3, 3.2), acyclovir should be administered promptly until HSE recurrence is excluded, while maintaining a high index of suspicion for an underlying autoimmune etiology. The Panel agreed that if autoimmune encephalitis is confirmed after HSE, immunotherapy should be used in a similar way to idiopathic/naive NMDARE.^e9,e10^

The Panel acknowledged that although immunotherapy is the therapeutic priority to treat the underlying disease, symptomatic management (such as antiseizure medications) is equally important (Table 3, 3.3). However, symptom management may be challenging and requires multidisciplinary expertise.^e11^ As stated in the recommendations, there was consensus on a preferred list of medications found to be useful in the treatment of behavior agitation and dyskinesia (full list of medications considered is detailed in eAppendix 1, links.lww.com/NXI/A530). Caution was also drawn to the observation that the use of antipsychotics in pediatric NMDARE may worsen dyskinesia or induce a neuroleptic malignant syndrome.

Although paraneoplastic etiology is rare in prepubertal children and in boys,^2,9,e12^ oncologic searches for ovarian teratoma (and neural crest tumors in children aged <5 years) are mandatory in all children with NMDARE, should be performed early, and be completed in the first days-weeks after admission (Table 3, 3.4). Ultrasound or MRI of the abdomen and pelvis and CT or MRI of the chest are the recommended imaging modalities, and collaboration with local oncologists and radiologists will help guide the need for additional studies (e.g., PET scan) to optimize diagnostic yield in patients with severe disease or a failure to improve. The timely identification of a tumor and its subsequent removal may improve the outcome considerably, although the prognosis also depends on the type of tumor.^2,e12^ The Panel reached agreement on oncologic searches that should be performed in all patients, both at baseline and in patients who fail to improve or relapse, with particular focus on postpubertal females in whom ovarian teratoma screening and longitudinal surveillance for ovarian teratoma should be strongly pursued.

Although not the main aim of this consensus document, the Panel acknowledged that adequate rehabilitation after the acute phase of NMDARE is essential and may improve outcomes. We strongly support the need for rehabilitation to be provided in a center familiar with rehabilitating young people with acquired brain injury such as encephalitis or traumatic brain injury, acknowledging that improvements may continue for up to 24 months. Rehabilitation often includes focus on cognitive and behavioral problems (including executive dysfunction and fatigue) post-NMDARE.

In view of the relative rarity of this condition, any recommendation or guideline for the treatment of pediatric NMDARE is inevitably based on limited evidence; therefore, this document should be intended as a recommendation meant to provide guidance rather than absolute rules, and it should not be used to prevent access to therapies if these are recommended by a patient's physician. Moreover, by putting together international experts from very different settings, the present work highlighted heterogeneity in the management of this condition. The differences stimulated discussion and reflection, and there was still consensus around most aspects of pediatric NMDARE treatment. Although the experts included people with broad international expertise, the opinions remain vulnerable to anecdote and potential bias related to referral of complicated or atypical patients.

Despite these limitations, we strove to create an international consensus-based recommendation aimed at supporting the clinician in the treatment of pediatric NMDARE, with a dedicated global approach for all health care settings. We hope that with the aid of recently released diagnostic criteria,^26,27^ the present treatment recommendation may contribute to a more systematic approach, resulting in more comparable data internationally, which may generate better quality evidence. Nonetheless, there are still major unresolved issues, which should represent the focus of future research.

## Glossary

HSE: herpes simplex virus encephalitis
IQR: interquartile range
IgG: immunoglobulin G
IVIg: IV immunoglobulin
NMDARE: NMDA receptor antibody encephalitis
TPE: therapeutic plasma exchange
